# Dynamics in implementing the Good Financial Grant Practice standard across three African universities: an Indigenous realist evaluation

**DOI:** 10.1186/s12961-025-01343-7

**Published:** 2025-05-26

**Authors:** Meshack Nzesei Mutua, Ferdinand C. Mukumbang

**Affiliations:** 1 Department of Organisational Psychology, University of Cape Town, Rondebosch, Cape Town, 7701 South Africa; 2 Department of Global Health, School of Public Health, University of Washington, Seattle, WA USA

**Keywords:** GFGP assessment, Institutional due diligence, Finance and grants management capacity, Indigenous realist evaluation

## Abstract

**Background:**

Research institutions must demonstrate the capability to efficiently and effectively manage external funding. The Good Financial Grant Practice (GFGP) was developed and operationalized as a capacity assessment and improvement tool and has been used by funding partners to assess and improve grantee institutions’ financial and grants management capacity. However, little is known about the effectiveness of the GFGP process. We conducted an Indigenous realist evaluation to examine how the GFGP works, why, for whom and under what circumstances to strengthen African institutional finance and grants management capacity.

**Methods:**

A multicase realist evaluation study design was employed. In total, three African universities (cases) of varying sizes were studied; 15 realist-informed qualitative interviews were conducted with research support staff, finance and grants personnel, principal investigators (PIs) and programme-level staff to test an initial programme theory. To test the theory, we applied retroductive theorizing and the context–mechanism–outcome (CMOs) framework. A realist-informed thematic analysis was employed to identify experiential, inferential and dispositional themes necessary for generating CMOs.

**Results:**

We found mechanisms that can enhance (commitment, motivation, awareness and empowerment) or limit (fear, frustration and resentment) the adoption of GFGP in different institutional contexts. Where an institution has an inefficient grants management system, fear of losing funding results in the nondisclosure of the inefficiency of the grants management system, and consequently, the inefficiency remains unresolved. Where the institution has a small external funding base but has an efficient centralized finance and grants management system, the staff are motivated and better aware of the grant processes, leading to the completion of the GFGP process and thus resulting in the review and update of the institution’s grants management policies. Where the institution has a large external funding base and has undergone participatory due diligence and audits by other international funders, the staff may feel frustrated and resent, causing the team to push back on the so-called unrealistic recommendations and expectations.

**Conclusions:**

A participatory/consultative approach to the GFGP process can ensure context-sensitive engagements and recommendations and promote stakeholder buy-in. Additional resources should be provided to address the identified financial and grants management capacity gaps as necessary.

## Introduction

Large inequities exist in global health research grants/funding tableau between low- and high-income countries [1]. Funding partners in high-income countries (HICs) have thus been called on to proactively address inequities in their approach to research funding and commit more direct funding to researchers, particularly in low-and middle-income countries (LMICs) [2, 3] to promote locally-led scientific knowledge. However, to make such commitments for direct funding, there is an indisputable need for accountability and transparency in grants management, which requires demonstrable finance and grants management capacities by research institutions [4–6].

The Good Financial Grant Practice (GFGP) Standard is an innovative tool for enhancing institutional finance and grant management capacity [7]. The GFGP Standard is designed to help institutional teams self-assess against set criteria and identify and address capacity gaps to inform their management actions [7, 8]. Launched in 2018, the GFGP Standard was developed by the African Academy of Sciences (AAS) in partnership with the African Organization for Standardization (ARSO), which published the Standard (ARS 1651). Multiple partners financially supported the initiative, including the Wellcome, UK Research and Innovation (UKRI), UK Department of Health and Social Care (UKDHSC), the IKEA Foundation, the European and Developing Countries Clinical Trials Partnership (EDCTP) and others collaboratively involved, including the African Union, the Foreign, Commonwealth and Development Office (FCDO) and the New Partnership for Africa’s Development and Coordinating Agency (NEPAD) [8, 10].

**Fig. 1:**
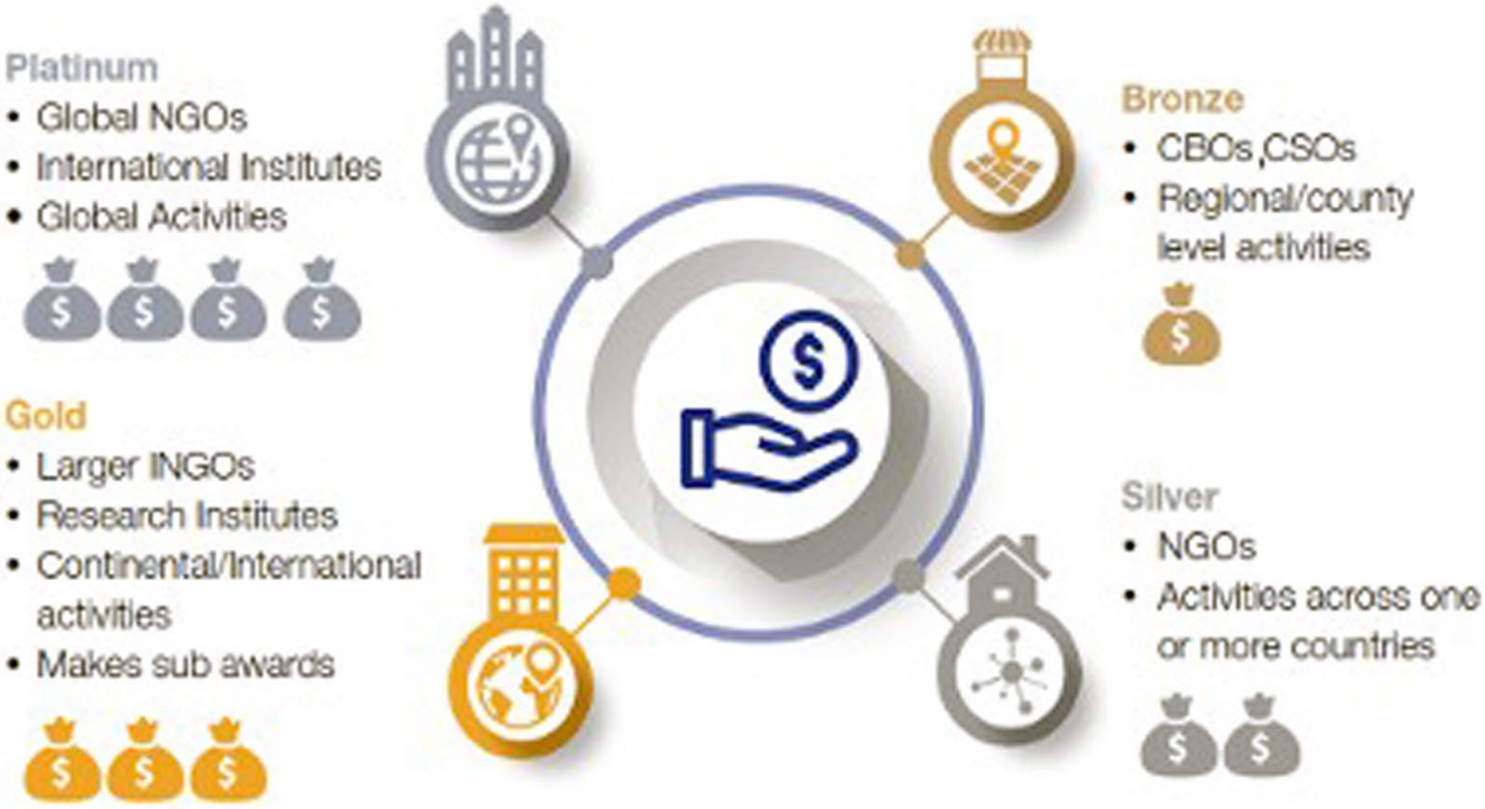
The Good Financial Grant Practice four-tier system (Source: Global Grant Community [9])

The GFGP Standard is implemented under the Global Grant Community (GGC) platform, which comprises:

- An online self-assessed precertification scheme (completed by prospective grantees) comprising more than 300 requirements cutting across four practice areas: institutional financial management, human resources, procurement and governance. The prospective grantee should complete between 70 clauses (bronze) and 300 clauses (platinum) and upload necessary supporting procedural (bronze), process (silver) and policy (gold/platinum) documentation.
- Network of audit firms licensed to undertake audits for certification. The audit firms review and audit the completed GFGP forms to verify the information and generate a report rating the grantee’s risk levels (i.e., high, medium or low) on the basis of the existing structures, policies and processes. The AAS uses the report to inform funding decisions and determine the support grantees need. If an organization is rated low risk, the grant is disbursed, while if rated high or medium risk, recommendations are made on areas of improvement that the institutions need to address before they can receive the grants.
- The GFGP Certification is a four-tier compliance scheme comprising the bronze (lowest tier), silver, gold and platinum (highest tier). The certification depends on the scale and complexity of funding and the size of the grantee institution (see Fig. 1). The assessment outcomes guide potential grantees to improve their finance and grant management capabilities and move up the tiers.

The GFGP Standard was established for research, government, nongovernment and academic institutions of any size to assess how effectively they can manage funding and/or their compliance with the Standard. The GFGP Standard aims to benefit the Global North and South institutions. The GGC platform allows (a) grantees and grantors to share best practices in financial and grant management, (b) funding partners to request prospective grantees to comply with the Standard as part of preaward due diligence and (c) grantees to use the Standard as a blueprint to improve their processes for grant management capabilities. The due diligence process is anticipated to be streamlined and strengthened while promoting trust and transparency in using funds to ensure efficiency and greater impact [10]. As a requirement, all prospective grantees undergo the GFGP assessment as part of due diligence before receiving funding from the AAS.

The GGC platform estimates that more than 250 organizations across 45 countries globally have completed the precertification assessment [10]. Despite the increasing interest in and implementation of the GFGP Standard [8–11], evidence on how and why the Standard works to improve grants management practices is scarce. Using the African Research Initiative for Scientific Excellence (ARISE) programme as a real-world case, this study applied an Indigenous realist evaluation to investigate how, why, for whom and under what conditions the GFGP works to strengthen research institutions’ finance and grants management capacity.

### Indigenous realist evaluation

Realist evaluation is a theory-driven approach that first emerged in a Pawson and Tilley [12] seminal work. Realist evaluation applies the context (C), mechanism (M) and outcomes (O) configuration framework to determine what works, how, why, for whom and under what circumstances [12, 13]. It involves engaging various programme stakeholders and participants to develop, test and refine theories about how and why a programme works. Renmans et al. [14] recently argued that the principles and practices that underpin realist evaluation have great potential to contribute to the decolonization of the global health endeavour. An Indigenous realist evaluation integrates the Indigenous research principles [15] for a more sensitive or responsive engagement with Indigenous and/or formerly colonized contexts. The nine Indigenous research principles include relationality, responsibility, reverence, reciprocity, respectful representation, reflexivity, responsivity, rights and regulation and decolonization [15]. A paper that discusses how the Indigenous-inspired realist evaluation can be operationalized in the context of health research capacity strengthening has been published elsewhere [16].

While Mukumbang and colleagues [17] argue that the realist evaluation approach blends critical and scientific realism principles, Mutua and Nakidde [16] posited that the Indigenous realist evaluation would be informed by both the realism and postcolonial Indigenous paradigms. The postcolonial Indigenous paradigm provides an additional lens through which an evaluator can explore and understand power dynamics, ensure ethical research conduct and promote the transformative power of evaluations [15, 18]. An Indigenous realist evaluation approach addresses the question of what works, how, why, for whom and under what circumstances and ensures that the participants’ voices are elevated and the social injustice and inequities addressed [16]. The Indigenous lens seeks to promote epistemic justice by fostering ethical conduct, centring the voices of the programme beneficiaries and generating evaluation that has real use to policy and practice [16].

### The GFGP assessment under the ARISE programme

The ARISE programme is a 5-year initiative (2022–2026) funded by the European Commission (EC) and jointly implemented by the African Academy of Sciences (AAS) and the African Union (AU). The programme has three objectives:

- Objective 1: strengthen the capacities of the emerging African research leaders committed to a research and teaching career in Africa,
- Objective 2: strengthen institutional research management and support systems for research to thrive and
- Objective 3: support the generation of cutting-edge research that will contribute towards transforming lives in Africa.

Through the ARISE initiative, African principal investigators (PIs) who have demonstrated the potential to become research leaders are supported in implementing locally relevant and locally led research projects. With funding support of up to €500,000 and hosted in an African research institution or university, the PIs are expected to lead the conceptualization and implementation of research projects exploring a wide range of research questions across different thematic areas such as health and climate change. Regarding institutional capacity strengthening (*Objective 2* above), the host institutions underwent the GFGP assessment process intending to strengthen their finance and grants management capacity.

The GFGP precertification self-assessment for the ARISE programme was completed between April and November 2022. The host institutions completed the online assessment, and then audit firms conducted desk reviews to verify and validate the assessment results. The aim was to determine whether the host institutions could host the ARISE grants and understand the existing financial and grant systems, processes and practices. If the host institution did not have adequate capacity (indicated by a high-risk rating and a score of < 60%), then the ARISE funding could not be disbursed to the institution.

To help contextualise the results, first, we present a summary of the GFGP assessment findings across the three cases in Table 1. The findings include the risk rating score, improvement areas and the auditors’ recommendations.

**Table 1:** Summary of the GFGP assessment results

Case	Risk score	Areas of improvement	Recommendation
Case A	69%	Subgrantee management, contract management, grant management and compliance and audit	Funding decisions should be based on assurance from the host institution on how the identified risks will be addressed
Case B	99%	Income management	The institution’s capacity is adequate
Case C	94%	Audit and risk management	The institution’s capacity is adequate

*Rating scale: *< *39%* = *critical risks; 40–59%* = *high risk; 60–79%* = *medium risk and *> *80%* = *low risk*

The assessment reports included the recommendations that the (i) Case A institution has been rated medium risk, and the partner should receive assurance on how the risks will be mitigated before disbursing the ARISE funding, (ii) Case B institution has been rated low risk and has adequate capacity for managing the ARISE funding and (iii) Case C institution has been rated low risk and has adequate capacity for managing the ARISE funding.

We started by eliciting an initial programme theory (IPT) as part of the realist evaluation. A programme theory comprises a collection of statements describing a specific programme, clarifying the reasons, for whom and the circumstances under which the programme’s effects happen. Developing an IPT – a set of assumptions of how the programme is expected to work – is the first step in a realist study [19]. We formulated an IPT on the GFGP assessment based on reviewing the ARISE programme documents, focus group discussions with the programme stakeholders and reviewing published literature. The IPT was framed as shown in ***Box 1*** below.

Box 1: initial programme theory IF there is buy-in from the research leaders (C), there is a system in place to support the receiving of foreign grants (C2), there are adequate resources to address capacity gaps (C3) and GFGP assessment identifies [host] institutional gaps (Intervention),

THEN the institutional finance and grants management systems, policies and practices would be improved (O)

BECAUSE the finance and grants team will become aware (M1) of their capacity gaps and be motivated (M2) to address them.

In this paper, our goal was to test and refine the GFGP assessment IPT to establish how and why the GFGP has worked to strengthen the institutional finance and grants management capacity and under what conditions.

## Methods

A multicase study evaluation design (involving three case studies) was employed. The case study design allows the investigation of a “phenomenon within its real-life context, especially when the boundaries between phenomenon and context are not evident” [20]. Case study design has been widely applied in realist evaluations [21–24]. In our study, the selection of the three cases was based on the nature of the research, the size and structure of the host research institution and the economic status of the host country (which was hypothesized to influence the government’s budgetary allocation for health research) (Table 2). Our three case studies constituted innovation-based, policy-based and laboratory-based research projects. The case studies were sequentially conducted to ensure that insights from one case study were applied in the subsequent one.

**Table 2:** Characteristics of the three cases

Characteristics	Case A	Case B	Case C
Nature of the research project	Innovation	Policy-based	Laboratory-based
Host institution	Research-intensive public university	Teaching-based private university	Teaching- and research-based public universities,
Institutional structure	Large, hierarchical and decentralized	Small, nonhierarchical and centralized	Medium-size, hierarchical and decentralized
Country’s economic status	Upper-middle income	Low income	Low income
Estimate total funding at the host institution (USD)	95,000,000	700,000	33,000,000
Percentage of the ARISE funding	0.6%	95%	1.5%

Both within-case and cross-case analysis and synthesis were carried out [25]

### Data collection and analysis

The aim of the data collection process in realist evaluation was to verify, validate or refute our theorized inner workings of the GFGP assessment (as captured in the IPT – see ***Box 1*** above) by eliciting information from the programme participants based on their experience of how the GFGP works.

Realist-informed qualitative methods were employed in the study. Participants were purposively selected on the basis of their experience with the programme, specifically, the GFGP process. Although the study targeted a wide range of participants, questions on the GFGP Standard were answered by only 15 [16] participants, including the PIs and the finance and grants and research support personnel across the three cases. See Table 3 below. Other participants (for example, collaborators and PIs’ mentors and students) did not comment on the GFGP assessment [theory], citing that they were not involved in the assessment process, and they were thus excluded from the sample. Although the sample size may seem small, the goal of qualitative sampling in realist research is to obtain information-rich participants. From a realist perspective, therefore, our sample size was sufficient to inform the development of the generative causal explanations of how the GFGP process works across contexts. The targeted staff were involved in the GFGP assessment process, and their voices counted because the programme theories were formulated on the basis of their experiences.

**Table 3:** Summary of data collection and participants

Data collection method	Total
In-depth interviews with PIs	3
In-depth interviews with the research support and finance and grants staff	8
Key informant interviews with programme-level partners	4

The interview questions were designed to elicit information about context (Cs), mechanism (Ms) and outcomes (Os) that were related to the GFGP process [26] – see Table 4 for the definition of context, mechanism and outcomes. The realist interviewing technique was applied. In this way, the initial programme theory was the subject of discussion and the basis for gathering relevant evidence needed to clarify, modify, approve or discredit the IPT [26]. Where necessary, probing questions were appropriately used to elicit deeper information about relevant context conditions, mechanisms and outcomes.

**Table 4:** Definition of context, mechanism and outcome

Term	Definition
Context	Context includes the key aspects of the physical, social, cultural and environmental conditions beyond the parameters of the formal programme architecture that have the causal impact of an intervention [27]
Mechanism	Mechanisms relate to the underpinning generative force that includes mental processes such as reasoning, motivation, incentives and cognition or emotions among stakeholders and beneficiaries that lead to outcomes [27]
Outcome	Outcomes are “either intended or unintended and can be proximal, intermediate, or final” [28]. It relates to changes to knowledge, attitude, skills, intention or behaviour generated by an intervention, programme or policy

In data analysis, we took a realist approach to thematic analysis, which was achieved by formulating experiential, inferential and dispositional themes [29]. As suggested by Pawson [30], we started the data analysis process at the IPT development phase by generating CMO configurations. Retroductive theorizing informed the overall data collection approach guided by the CMO heuristic, and abductive reasoning (both inductive and deductive logic) was applied to the various data sources [31]. Using the CMO heuristic tool, we identified excerpts denoting mechanisms activated by the so-called GFGP process across the three cases to generate intended or unintended positive or negative outcomes. Where too many contexts and mechanisms were identified, we abstracted the key elements through accentuation, highlighting the most prominent contexts and mechanisms [32] to prioritize CMO elements with greater explanatory potential.

## Results

We identified mechanisms that speak to the relevance and perceived usefulness of the GFGP. A total of four case-based theories emerged from the evidence, including (1) fear, commitment and motivation, (2) motivation and awareness, (3) frustration and resentment and (4) empowerment and commitment. For each programme theory, we constructed if… then… because… statements and figures representing what works, for whom, how and why and under what conditions to improve the translation of the evidence [33].

### Theory 1: fear, commitment and motivation (Case C)

The participants (Case C) highlighted that their current paper-based financial and grant management system was inefficient (C). The team reported that their primary institutional need was to install a digitalized/automated grant management system, which required large initial capital. Completing the GFGP assessment as a precondition for funding triggered so-called fear of losing the grant opportunity (M1) among the finance and grants team, thus withholding information about their inefficient finance and grants management system (outcome). During interviews, the grants staff indicated that they had a grant management system in place but did not disclose that it was paper-based, which is characterized by inefficiency in the finance and grants management process. The fear (M1) triggered in the context of an inefficient grants management system resulted in nontransparency in the capacity gaps (O1) and, consequently, the inefficiency not being addressed by the ARISE (O2).

…we are still using the paper-based grants management system, which is quite inefficient [C1]. We don’t have a dedicated and automated grants management system that can track all the processes of the grant cycle. That one, at the moment, is a very critical gap. …we only indicated that we have a grant management system but did not disclose that it is not an automated computer-based system. I think there was fear that we could lose the funding, having come so far in the application process [fear] [M1], so the person completing the assessment omitted that detail (interview, grants manager, Case C).

As a Unit, we have been committed to supporting our faculty staff with the finance and grants matters [M2] to ensure they successfully implement their research projects. Despite our shortcomings with our finance and grants management system, we are committed as a team to providing our researchers with the support they need [M2], and when we were called on to complete the GFGP process, we did that. …I think we were motivated knowing that the GFGP assessment was the last thing we needed to do [motivation] [M3] before Dr. [PI] could receive the ARISE funding (interview, head of grants unit, Case C).

The finance and grants team also highlighted that, notwithstanding the shortcomings of their grants management system, the team was committed (M2) and motivated (M3) to provide the necessary finance and grants management support to their early career researchers. The commitment and motivation led to the completion of the GFGP process.

**Fig. 2:**
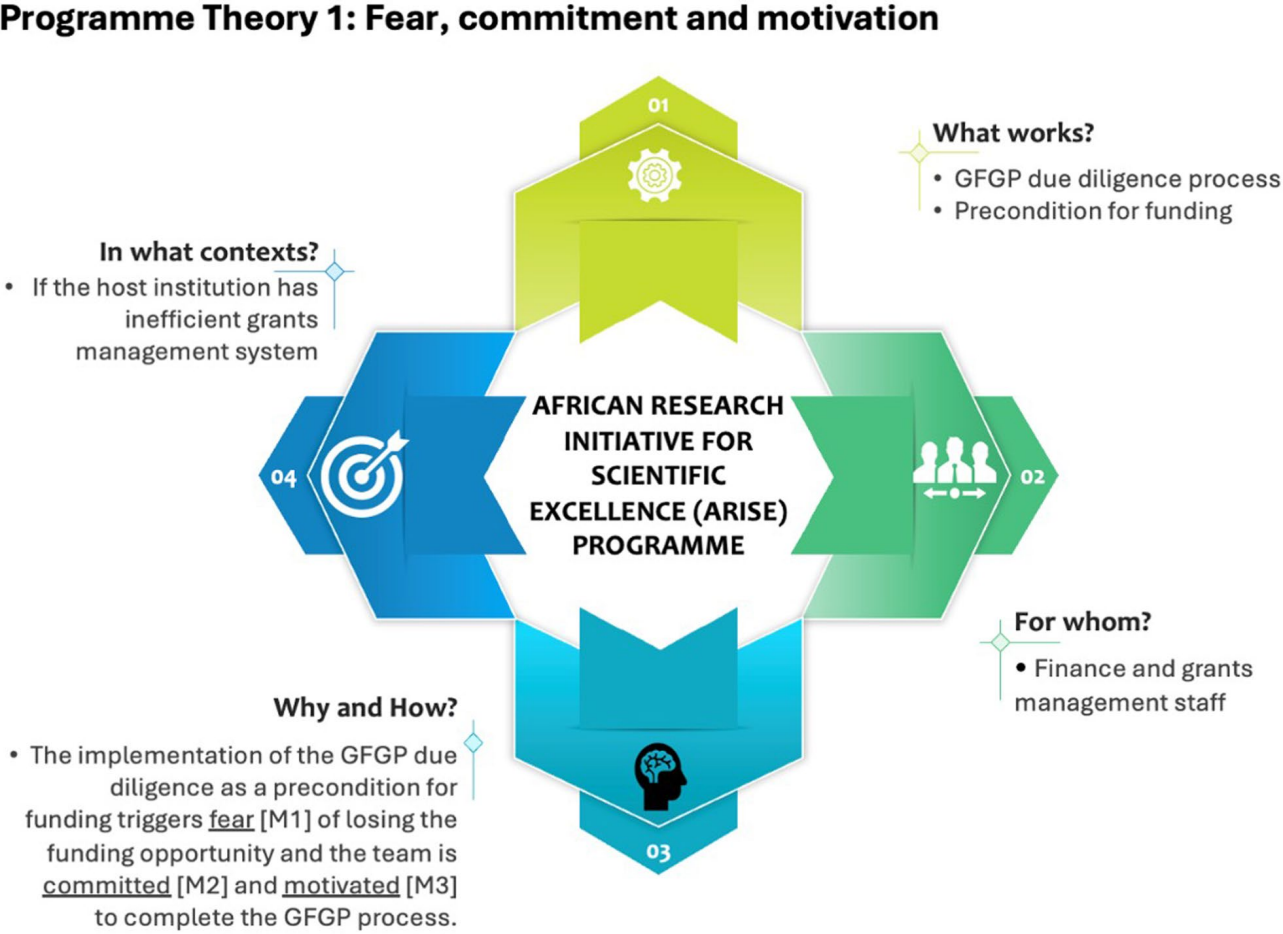
Programme theory 1

Programme theory 1 has been summarized – see Fig. 2 above. This theory can be represented using the if… then… because… statement shown in Box 2 below.

Box 2: programme theory 1 IF the host institution has inefficient grants management system (C), and the GFGP due diligence process is a precondition for funding (I),

THEN there will be nontransparency (inefficiency of the grants system will not be disclosed) in the completion of the GFGP assessment (O1), and inefficiency will not be addressed (O2)

BECAUSE the fear of losing the funding opportunity (M1) is triggered and the grants team is committed (M2) and motivated (M3) to complete the GFGP process.

## Theory 2: motivation and awareness (Case B)

The institution’s (Case B) external funding base was identified as an important context condition. The participants highlighted that the institution managed relatively small external funding, which somewhat influenced their approach to the GFGP process. This point was also corroborated by an analysis of the estimated external funding base and the percentage of the ARISE funding across the three cases. The analysis shows Case B had the least funding base (Table 2). The ARISE grant was their biggest external funding (representing 95% of the total external funding) at the time of the evaluation. The finance and grants personnel highlighted their motivation to ensure that their management system is good and that their research staff secures funding. By going through the GFGP process, the finance and grants team could reflect and take stock of their institutional policies and processes, thus increasing their awareness. This notion is captured in the quotes below.

…we are still a small institution with a relatively small funding base (C1). So, everything is centralized, including the finance and grants management system and processes (C2). The ARISE grant is currently our biggest external funding. …for us, the GFGP assessment kinda motivated us to complete the process because we’d like to see our researchers securing more funding [motivation] [M]. …when we completed the process, we realized that the process had helped us to reflect and take stock of our policies and processes, which improved our awareness [M2] of our institutional policies and process [O]. (interview, finance, Case B).

We are a small university, and I think you saw the size of our premises when you came in this morning. So, we do not have those complicated hierarchies in finance and grants management processes, but they are quite simple and efficient [C2]. Everything is very centralized [C2] (interview, research support, Case B).

The participants reported that in addition to the awareness (about their finance and grants management policies and processes) gained following the completion of the GFGP process, the team also acted on some of the recommendations made by the GFGP audit team. Some actions included reviewing and updating the institutional procurement and grants management policies (O).

…as an institution, we have acted on some of the recommendations made by the GFGP. For instance, we have been able to review and update our procurement and grants management policies, and the board adopted the changes [O]. …the GFGP process has undoubtedly been useful to us (interview, finance, Case B).

**Fig. 3:**
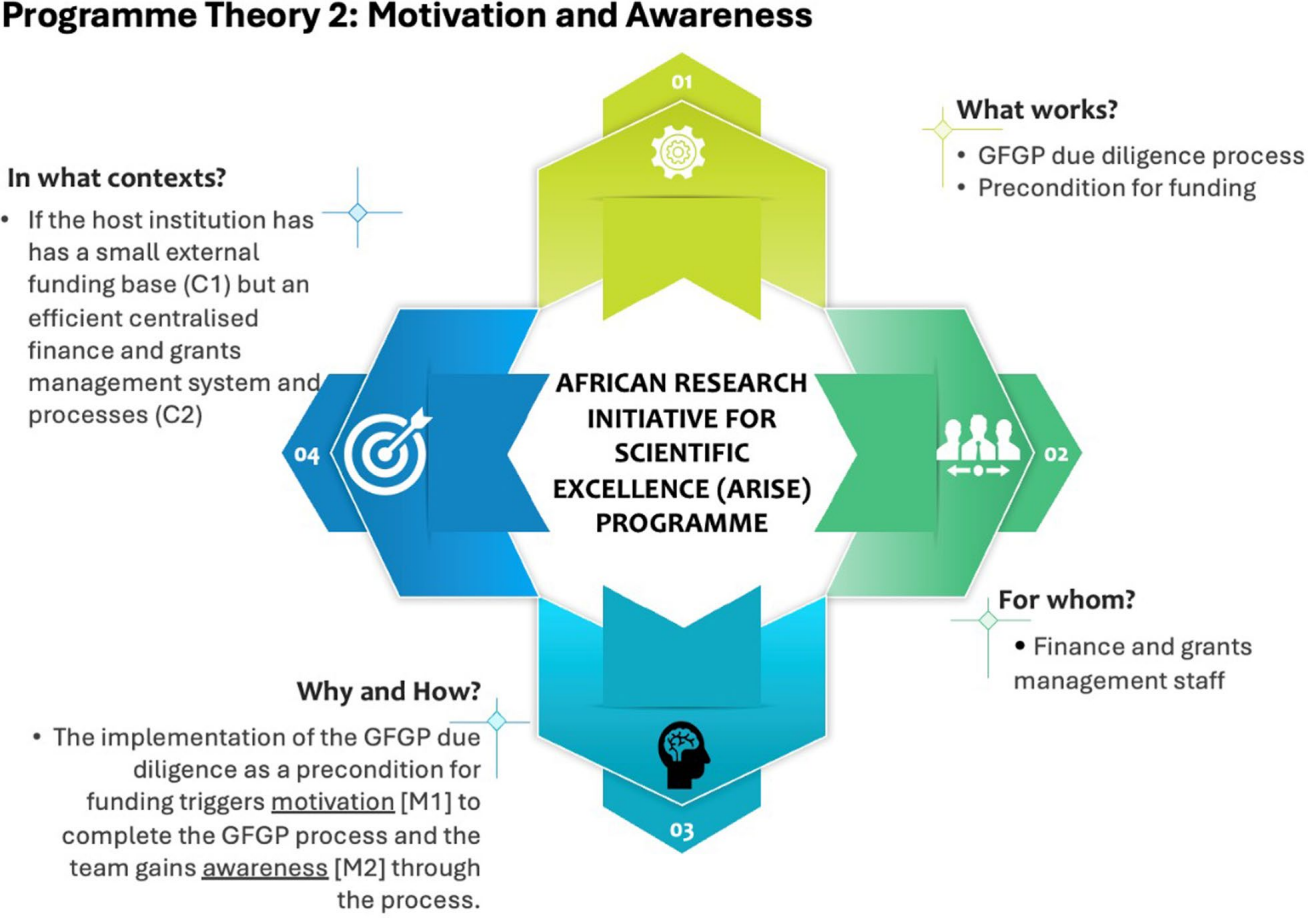
Programme theory 2

Programme theory 2 has been summarized in Fig. 3. This theory can be represented using the if… then… because… statement, as shown in Box 3 below.

Box 3: programme theory 2 IF the host institution has a small external funding base (C1) but an efficient centralized finance and grants management system and processes (C2), and the GFGP due diligence process is implemented as a precondition for funding (I),

THEN the procurement and grants management policies are reviewed and updated (O)

BECAUSE motivation (M1) to complete the GFGP process is triggered, and the finance and grants team gains awareness (M2) through the process.

## Theory 3: frustration and resentment (Case A)

The finance and grants team (Case A) reported that the institution managed large external funding and that the ARISE grant was just 0.6 per cent of its external research funding portfolio (Table 2). During interviews, the participants repeatedly mentioned the large external funding base, attributing the large external funding portfolio to the established institutional finance and grants management structures. The finance and grants team argued that the university had secured significantly large funding from internationally competitive funding schemes, a testament to the institution’s well-established institutional finance and grants systems and processes. Additionally, the team reported that the institution has undergone multiple due diligence and audits (conducted in a participatory manner) by other international funding partners, and no serious audit issues or queries have been raised. Frustration (M1) and resentment (M2) were triggered in both context conditions as the finance and grants team wondered why the nonparticipatory GFGP process could identify risk issues when other participatory due diligence and audits by funders did not raise any capacity concerns. Importantly, resentment (M2) was also triggered in the context of the institution’s large external funding base, particularly the implementation of the GFGP’s due diligence process as a precondition for funding. This idea is captured in the quotes below.

…if you look at our university with [∼ USD 95 million] enterprise, 90% is external …we get more than 90% of our research funding from external, and about 60% to 70% is foreign. ...we run a thousand plus projects at any time and multisites [C1]. …we also have the likes of [funding partners] who have heavily funded some of our research initiatives, and before they gave us the funding, they sent a team of audits down here. We worked together and had multiple discussion sessions, and they produced a report sensitive to our reality [C2]. I think we have been frustrated [M1] by the nonconsultative nature of the GFGP process and some of the recommendations that are not sensitive to our institutional context and reality. With all the funding we are managing, how can anyone question our capacity to manage grants? (interview, finance 1, Case A).

…I think the GFGP process has left the team [finance and grants team] with a feeling of resentment [M2], like wondering why they can be rated medium risk by the GFGP when they have been categorized as well capacitated by internationally competitive funding partners and entrusted with grants running into tens of millions of dollars. …the idea of the GFGP assessment does not sit well with the university, and particularly given the nonparticipatory manner in which it has been implemented [I], it has badly frustrated the team [M1] (interview, PI, Case A).

Interviews with the finance and grants team established that the GFGP audit team did not take time to understand the institutional model of research operations or hold consultation meetings with the finance team. The audit team’s lack of effort to understand the operational models of the university resulted in recommendations that did not consider the institutional context. Importantly, the finance and grants team pointed out that the failure by the ARISE to provide additional resources necessary for addressing the identified capacity gaps “frustrated” them (M) further. The frustration and resentment mechanism resulted in the GFGP recommendations being pushed back by the finance and grants team.

The problem I had with the audit company, which I found frustrating [M1], was that they didn’t listen to or speak to the [university] stakeholders. They sat behind a desk and came up with their report, in which they rated us as a medium risk, which, to me, was neither here nor there. …there are issues of resources and time, so getting an instruction from GFGP telling you that you need to do things this way before we give you the funding is not going to work [O]. …you need to understand that GFGP is a one-size-fits-all tool, and for it to work, the process needs to be participatory, the audit team needs to have a better understanding of the realities and needs of the institutions, consultatively identify areas of improvement, and working together, devise a plan on how to improve the areas and taking into account the available resources (interview, finance 2, Case A).

A review of the GFGP audit reports revealed that the audit team based their recommendations and conclusions primarily on the online self-assessment report (on the GFGP online portal) without meaningful engagement and consultation with the finance and grants teams.

For each of the grantee’s organization, we reviewed the details provided on the GFGP Online Assessment to determine completeness and compliance with the set Standard and identified any gaps in the information provided to enable us to reach the conclusion as required (Document 3, p.8)

“Under each area covered [Financial Management, Human Resource, Procurement and Governance], we reviewed the GFGP self-review report provided on the GFGP online web. After completing the review and assessing the support documents and responses provided, we [have] constituted a report indicating the gaps noted and indicated the recommendations that need to be implemented to improve the status in the organization operations as necessary” (Document 2, p. 3).

The reports did not indicate anywhere that the institutions were engaged in the due diligence process, which remained a critical gap in implementing the GFGP.

**Fig. 4:**
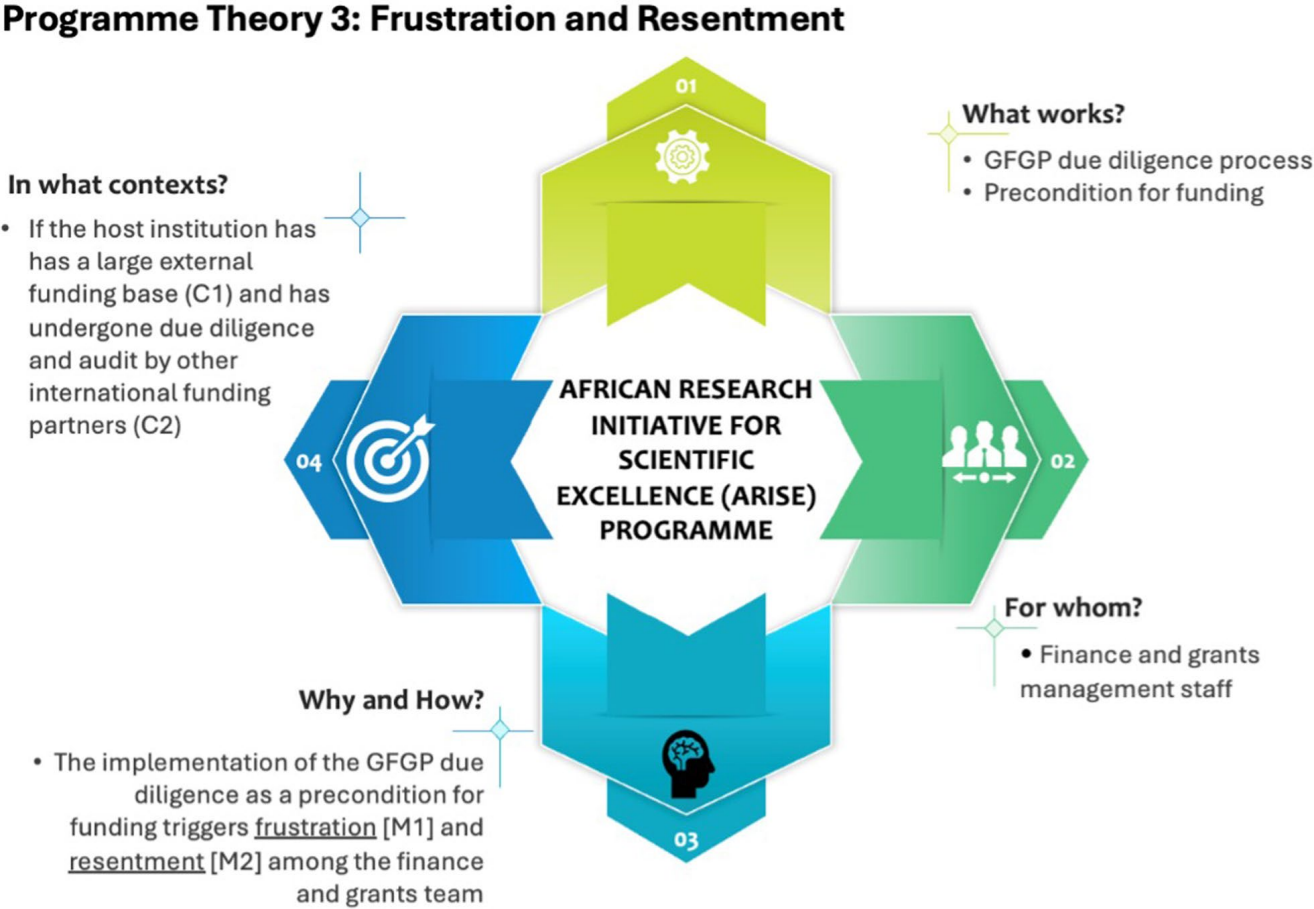
Programme theory 3

Programme theory 3 has been summarized (Fig. 4). This theory can be represented using the if… then… because… statement shown in Box 4 below.

Box 4: programme theory 3 IF the host institution has a large external funding base (C1) and has previously undergone participatory due diligence and audit processes by international funding partners (C2), and the GFGP due diligence process is implemented as a precondition for funding (I),

THEN the finance and grants team will push back unrealistic GFGP recommendations and expectations (O)

BECAUSE frustration (M1) and resentment (M2) will be triggered among the finance and grants team.

## Theory 4: empowerment and commitment (Case A)

A key context condition was identified as the robust and efficient decentralized finance and grants management system and processes (C1). According to participants, the robust finance and grants management system has effectively provided preaward financial support (for example, budget preparation for a grant application, the GFGP assessment) and postaward support and reporting (for example, preparation and submission of financial progress reports and support for regular audits) to faculty staff, including the ARISE research team. The robust and efficient decentralized finance and grants management system and process (C1) trigger empowerment (M1) of the finance and grants team to offer the support needed by the ARISE research team.

The faculty has well-established, robust and efficient finance and grants management policies and practices perhaps because the salaries of about 50% of the staff are directly funded from research grants [C1]. As such, we have a strong decentralized finance and grants management system and processes there including a dedicated team or preaward finance support, postaward finance support and reporting and that’s empowering for the team. They have the right tools and know what to do to effectively support our researchers [empowerment] [M1] (interview, finance 1, Case A).

…we are committed [M2] and, of course, well equipped to offer our support to our researchers from early career to senior researchers … we’re committed to going to great lengths to ensure that they get all the support they need throughout the preaward and postaward stages [M2]. … it’s situations like those that remind us of our commitment [M2], and we just go through it [GFGP assessment] for the sake of our early career researchers who yearn to grow in their careers (interview, finance 2, Case A).

The finance and grants team also highlighted that their commitment (M2) to effective and efficient finance and grants support to researchers meant they were open to improving their policies and processes. The finance and grants team reported developing their departmental key performance indicators (KPIs) on the basis of several contextually relevant recommendations made by the GFGP audit team. Consequently, this helped to improve their departmental plans. In this case, the robust finance/grants management system and processes and the institutional commitment (C2) have empowered (M1) and motivated (M2) the finance/grants staff to support researchers through the GFGP process, thus resulting in improved departmental planning (O).

I mentioned our commitment to supporting our faculty staff right from the pre-award grant process [M2] all through implementation and closing of grants and as an institution, we have remained committed even to improving how we do things. You might have heard of our frustration with the GFGP process, but that’s related to how it was implemented and some of the recommendations that are not context-sensitive or don’t consider our institutional resources and realities. But obviously we had a few areas that we could act on. …the process has helped us to identify some areas that need improvement, and we have developed some KPIs based on those findings. …it has improved our departmental plans [O] (finance 2, Case A).

…we have looked at what gaps are there that we need to solve and for our own KPIs, we looked at the recommendations and asked ourselves how we could address those gaps, for instance, in relations to how we approach subcontracting, and we ensured that the finance department’s KPIs are aligned [O]. …so that is how we have approached the positive stuff from the GFGP process. We ignore the not-so-positive ones, especially those not aligned with our institutional realities and operating model (finance 3, Case A).

The achievement of “improved departmental plans” (positive) outcome by the Case A team highlights the complexity of the GFGP process. The same team expressed frustration and resentment with the GFGP process (see programme theory 3 above). Therefore, it is evident that varied mechanisms can be simultaneously triggered, resulting in different outcomes within the same context.

**Fig. 5:**
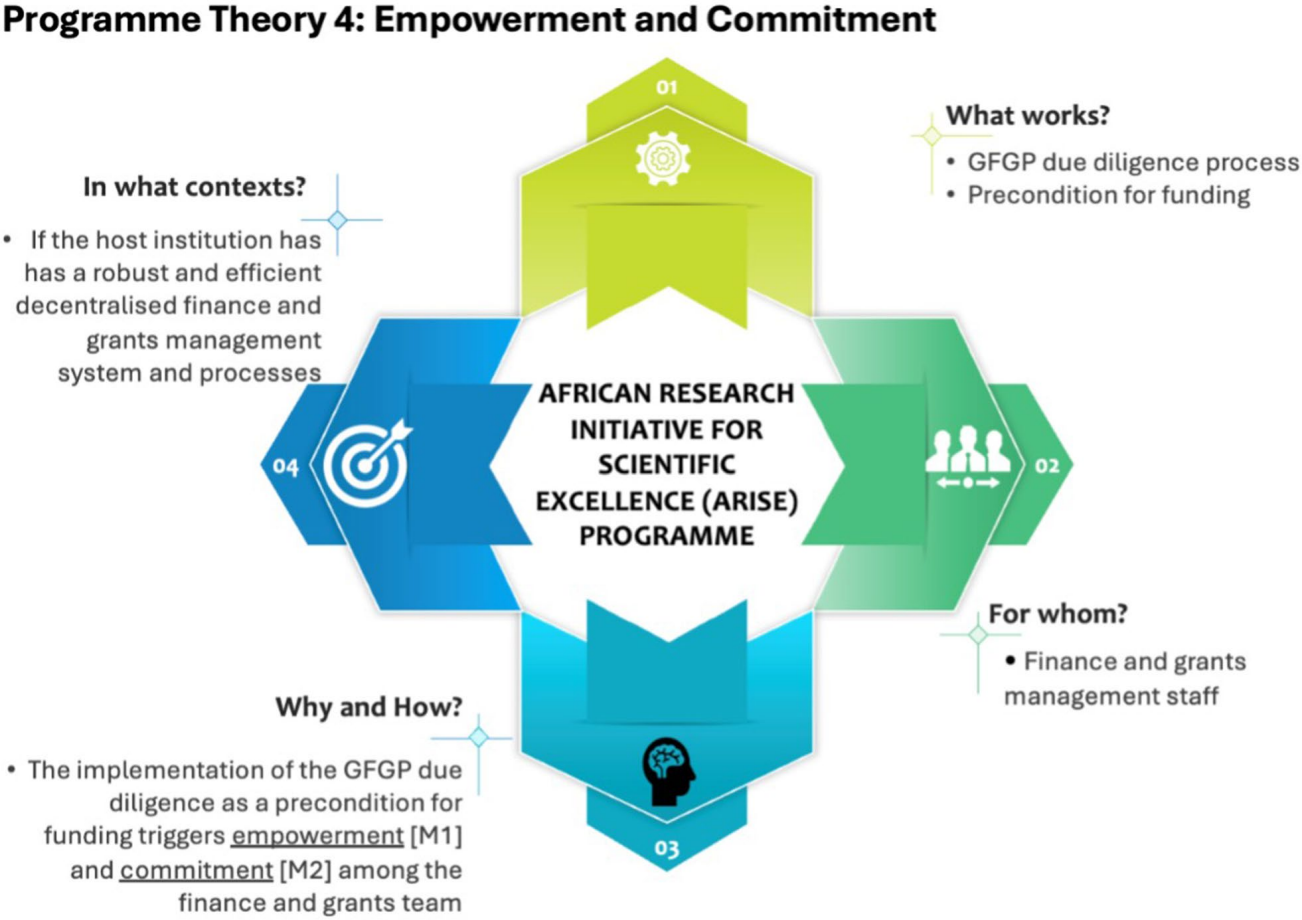
Programme theory 4

Programme theory 4 has been summarized (Fig. 5). This theory can be represented using the if… then… because… statement shown in Box 5 below.

Box 5: programme theory 4 IF the host institution has a robust and efficient decentralized finance and grants management system (C), and the GFGP is implemented as a due diligence process (I),

THEN departmental plans will be improved (O)

BECAUSE the finance and grants management team will be empowered and committed to support researchers through the GFGP process (M)

## Discussion

This study has established that the GFGP will work differently in different contexts to generate varied outcomes on grantee institutions’ financial and grants management capacity. Across the three universities, we identified mechanisms that can enhance (commitment, motivation, awareness and empowerment) or limit (fear, frustration and resentment) the adoption of GFGP in different institutional contexts. Where an institution has an inefficient grants management system, fear of losing funding results in the nondisclosure of the inefficiency of the grants management system, and consequently, the inefficiency remains unresolved. Where the institution has a small external funding base but has an efficient centralized finance and grants management system, the staff are motivated and better aware of the grant processes, leading to the completion of the GFGP process and thus resulting in the review and update of the institution’s grants management policies. If the institution has a large external funding base and has undergone participatory due diligence and audits by other international funders, the staff may feel frustrated and resentful, causing the team to push back on the so-called unrealistic recommendations and expectations. Notably, where the institution has a large funding base and a robust and efficient decentralized finance and grants management system, the staff are empowered and committed, completing the GFGP process and thus resulting in improved departmental planning.

Frustration and resentment in the context where an institution has a large external funding base and has undergone participatory due diligence and audits by other international funding partners, resulting in back-on GFGP recommendations, is characteristic of a power dynamic. The small research institutions keen to secure research funding may be less likely to critique the GFGP process for not being responsive to the institutional context. Harste et al. [8] argue that a power imbalance characterizes the due diligence process of GFGP, as the funding partners request information before the funds are transferred to the grantee, which puts an onerous burden on the grantees. On their part, the funding partner needs an assurance that the funding will be effectively managed by understanding the institution’s financial risk profiles [5]. It is not surprising that Stergiou and colleagues [34] argued that the GFGP process would be more useful in institutions characterized by high financial risk, and, interestingly, went on to highlight that the GFGP was not appropriate for the UK-based research organizations because of its heavy focus on finance (p. 9).

Commitment is a cross-cutting mechanism across almost all cases. The commitment of the finance and grants management teams to support their (early career) researchers makes it possible for them to complete the GFGP process even when they already have large external funding (Case A) or an inefficient finance and grants management system. Commitment to the provision of financial and grants management support has also been cited by Makokha et al. [35] as a critical mechanism. Besides the commitment identified in a specific context to generate positive outcome(s), other mechanisms are triggered, thus highlighting the complexity of the GFGP process. For instance, the achievement of “improved departmental plans” (positive) outcome while at the same time pushing back so-called unrealistic GFGP recommendations and expectations (see programme theory 3 and 4 above) demonstrates that multiple mechanisms can be simultaneously triggered, resulting in different outcomes within the same context.

The GFGP and its capacity strengthening objective are based on the understanding that capacity assessment can lead to capacity strengthening [36]. Jackson et al. [37] and Stergiou et al. [34] also argue that the GFGP could be deployed as a capacity-building tool to strengthen research institutions. Gonzalez et al. [38] and Miller et al. [39] have argued that capacity assessment can be turned into strategizing and decision-making tools for capacity strengthening. As an unintended positive consequence, capacity strengthening can be achieved by raising awareness of gaps, pointing out what actions must be taken to address the gaps and taking appropriate actions [36]. Although capacity assessment can lead to capacity strengthening [36], capacity strengthening that requires stakeholders’ actions beyond awareness creation will require additional funding resources. In their assessment of research management structures, Wallis and colleagues [40] highlighted that their programme provided institutions with limited funding to address several capacity gaps. This study established that there were no similar provisions in the ARISE programme, consequently frustrating the team. As evident in Case A, while some of the recommendations (from the GFGP audit process) formed part of the departmental key performance indicators (KPIs) and were integrated into departmental plans, the team disregarded other recommendations with resource implications. This perspective is consistent with Harste et al. [8], who pointed out that while some institutions may design effective implementation plans for addressing the recommendations made by the GFGP assessment, some institutions may lack the necessary resources to address the capacity gaps (S278).

Although the GFGP Standard has been described as a one-size-fits-all tool that can help improve institutional finance and grants management capacity [8], this study has established that the GFGP will work differently in different contexts to generate varied outcomes. Several institutions have centralized finance and grants management systems and processes, and others have them more decentralized, and, while some of them will self-assess against GFGP at an institutional (centralized system), others will self-assess at the departmental level [8]. Therefore, the GFGP process should consider the institution’s operational model (context) for the process to be effective and add value to the institution. In the words of a participant, “you need to understand that GFGP is a one-size-fits-all tool and for it to work, the process needs to be participatory, the audit team needs to have a better understanding of the realities and needs of the institutions, consultatively identify areas of improvement and, working together, devise a plan on how to improve the areas and taking into account the available resources”. Adopting a participatory or structured engagement of the institutional stakeholders throughout the due diligence process makes it possible for the due diligence process to yield a deeper and more meaningful impact [41].

This study has two key strengths. First, the study provides critical insights into how the GFGP works to strengthen institutional finance and grants management capacity under the ARISE programme. The Indigenous realist evaluation has provided a structured approach to understanding the complex interplay between context and mechanism that facilitates the generation of intended and unintended outcomes across the university contexts. The evidence is useful to policymakers and practitioners as it promotes a deeper understanding of the effectiveness of the GFGP and highlights areas for improvement, which – if acted on – can make the GFGP more responsive to the needs of the target institutions. Second, given the dearth of evidence/literature on the GFGP Standard and its effectiveness, this study significantly contributes to evidence-building. It adds to the few papers exploring a largely unexplored topic. Notably, given the centrality of the Indigenous realist evaluation approach in fostering ethical conduct, centring the participants’ voices and generating evaluation that has real use to policy and practice, the evidence generated in this study was disseminated to the ARISE programme partners and stakeholders, clearly highlighting what makes the GFGP process work across institutional contexts and what needs to be done differently to maximize its potential.

This study is not free of limitations. First, the interpretation of the results in this study should consider that the GFGP Standard was not implemented to the letter within the ARISE programme. While the GFGP process constitutes three phases (that is, online self-assessed precertification scheme, audits process and the GFGP Certification), this study established that the GFGP implementation under the ARISE programme did not include the certification phase. Additionally, the GFGP due diligence did not assess the three institutions against any of the four tiers (that is, bronze, silver, gold and platinum). None of the three GFGP audit reports referred to any GFGP tiers. Rather, only risk-rating scores and recommendations were made without indicating what GFGP tier each institution was assessed against. The full implementation of the GFGP Standard might involve activating different mechanisms across contexts, resulting in generating varied outcomes. Future (realist) studies should explore how the full implementation of the GFGP might work across contexts and what mechanisms would be fired to generate intended or unintended capacity outcomes.

## Conclusions

This paper provides practice-relevant evidence on how the GFGP works, why, for whom and under what conditions to generate positive or negative outcomes. The evidence emphasizes the need for the GFGP process to consider the context of the host institutions, consultatively work with the finance and grants management staff throughout the GFGP process and consider the existing institutional or programme resources required to address capacity gaps. Since institutions come in all shapes and sizes, the GFGP audit teams should strive to understand the uniqueness of each research institution and the operational model of each institution and determine how the GFGP process can add value to each specific institution (context). The GFGP is a one-size-fits-all tool; however, for it to be effective, its implementation process should consider the uniqueness of each institutional context and reality.

## Data Availability

All relevant data have been provided within the manuscript.

## Abbreviations

ARISE: African Research Initiative for Scientific Excellence
GGC: Global Grant Community
GFGP: Good Financial Grant Practice
CMOCs: Context–mechanism–outcomes configurations
